# ﻿*Paraphlomisanisochila* (Lamiaceae), a new species from Hunan, China

**DOI:** 10.3897/phytokeys.259.153345

**Published:** 2025-07-08

**Authors:** Ang Liu, Hai-Bo Chen, Fan Zhang, Ya-Ping Chen

**Affiliations:** 1 Central South University of Forestry and Technology, Changsha 410004, China Central South University of Forestry and Technology Changsha China; 2 Dao County Yueyan State Forest Farm, Yongzhou 425305, China Dao County Yueyan State Forest Farm Yongzhou China; 3 Key Laboratory of National Forestry and Grassland Administration on Plant Conservation and Utilization in Southern China, Guangzhou 510650, China Key Laboratory of National Forestry and Grassland Administration on Plant Conservation and Utilization in Southern China Guangzhou China; 4 University of Chinese Academy of Sciences, Beijing 101408, China University of Chinese Academy of Sciences Beijing China; 5 Institute of Plant Conservation, Hunan Botanical Garden, Changsha 410116, China Institute of Plant Conservation, Hunan Botanical Garden Changsha China; 6 CAS Key Laboratory for Plant Diversity and Biogeography of East Asia, Kunming Institute of Botany, Chinese Academy of Sciences, Kunming 650201, China Kunming Institute of Botany, Chinese Academy of Sciences Kunming China

**Keywords:** Evergreen broad-leaved forests, Lamioideae, Paraphlomideae

## Abstract

*Paraphlomisanisochila*, a new species from the subtropical evergreen broad-leaved forests of Hunan Province, southern China, is described and illustrated. Morphological comparisons indicate that *P.anisochila* is most similar to *P.seticalyx*, whereas molecular phylogenetic analyses based on a comprehensive sampling and two nuclear ribosomal DNA regions (ITS and ETS) place it as the sister species of *P.yingdeensis*. *Paraphlomisanisochila* is characterized by its unequal corolla lips, distinguishing it from all other *Paraphlomis* species. Additionally, it differs from *P.seticalyx* in lamina shape, petiole and calyx length, and from *P.yingdeensis* in plant indumentum, calyx tooth apex, and corolla color.

## ﻿Introduction

Evergreen broad-leaved forests (EBLFs) are shaped by a monsoon-dominated climate and represent a distinct biome with exceptionally high biodiversity in East Asia, including southern China, Japan, and the Korean Peninsula (Zhang et al. 2024). *Paraphlomis* (Prain) Prain (Lamiaceae, Lamioideae) is a characteristic component of EBLFs, as the majority of its species are confined to this biome, with only a few extending into tropical Asia (Wu and Li 1977; Li and Hedge 1994; Chen et al. 2021; Yuan et al. 2024). As a member of the recently established tribe Paraphlomideae, *Paraphlomis* consists of perennial, stoloniferous herbs distinguished by simple hairs, axillary verticillasters, and an actinomorphic, tubular to obconical calyx with five lobes (Bendiksby et al. 2011; Chen et al. 2021). The corolla is bilabiate, with an entire upper lip and a three-lobed lower lip, while the mericarps are obovoid or triquetrous-oblong, either glabrous or hairy (Bendiksby et al. 2011; Chen et al. 2021). Compared to earlier floristic treatments (Wu and Li 1977; Li and Hedge 1994), the number of recognized *Paraphlomis* species has doubled due to a recent surge in newly described taxa and the taxonomic amalgamation of *Matsumurella* Makino and *Ajugoides* Makino into the genus (Yuan et al. 2024). Under its current circumscription, *Paraphlomis* now comprises 49 species and six varieties.

In this study, we describe a new species of *Paraphlomis* from the EBLFs in Hunan Province, southern China. We confirmed its taxonomic status and systematic placement by carrying out comprehensive morphological comparisons and molecular phylogenetic analyses based on a sampling of more than 85% taxa of the genus and two nuclear ribosomal DNA (nrDNA) sequences. The new species, *P.anisochila* Y.P. Chen, A. Liu & F. Zhang, is formally described and illustrated below.

## ﻿Materials and methods

### ﻿Morphological comparisons

Morphological comparisons of the putative new species with other *Paraphlomis* species were initially conducted through a review of the protologues of all validly published names and other relevant taxonomic literature (e.g., Wu and Li 1977; Li and Hedge 1994). Additionally, herbarium specimens, particularly type specimens, were examined across multiple herbaria, including BM, CDBI, CSFI, E, GXMG, GXMI, HAST, HIB, IBK, IBSC, JIU, JJF, K, KUN, KYO, MW, NAS, PE, SM, SZ, TI, and WUK (Thiers 2025). Photographs of living *Paraphlomis* plants available from the Global Biodiversity Information Facility (GBIF, https://www.gbif.org/) and the Plant Photo Bank of China (PPBC, http://ppbc.iplant.cn/) were also reviewed.

The new species was described based on field observations and herbarium specimen examinations. Morphological descriptions follow the terminology outlined by Li and Hedge (1994). Distribution data were compiled from herbarium specimen records, our own collections, and additional records obtained from GBIF and PPBC. Based on these data, a distribution map of the new species and its closely related species was generated using ArcGIS v.10.8.

### ﻿Phylogenetic analyses

Only the nuclear ribosomal internal and external transcribed spacers (ITS and ETS) were selected to determine the systematic placement of the putative new species, as plastid markers have previously failed to resolve phylogenetic relationships within *Paraphlomis* and have shown significant conflict with nrDNA trees and morphological data (Chen et al. 2021; Yuan et al. 2024). The ingroup comprised 44 species and four varieties of *Paraphlomis*, while *Phlomis* L. and *Phlomoides* Moench were included as outgroups following Chen et al. (2021). With the exception of one accession of the putative new species and two accessions of *P.seticalyx* C.Y. Wu, all sequences were retrieved from previous studies and downloaded from GenBank, with accession numbers provided in Appendix 1.

Genomic DNA of the putative new species and *P.seticalyx* was extracted from silica-gel-dried leaves using a modified cetyltrimethylammonium bromide (CTAB) method (Doyle and Doyle 1987). Polymerase chain reaction (PCR) amplification and sequencing of the two nrDNA regions followed the protocols described by Chen et al. (2021). Raw sequences were assembled using Geneious v.11.0.3 (Kearse et al. 2012), and sequence alignment was performed with MUSCLE (Edgar 2004) as implemented in MEGA6 (Tamura et al. 2013). The two nuclear sequences were then concatenated for phylogenetic analyses.

Phylogenetic trees were reconstructed using partitioned Bayesian inference (BI) and partitioned maximum likelihood (ML) methods, both implemented via the Cyberinfrastructure for Phylogenetic Research Science (CIPRES) Gateway online server (http://www.phylo.org/; Miller et al. 2010). BI analysis was conducted with MrBayes v.3.2.7a (Ronquist et al. 2012), while ML analysis was performed using RAxML-HPC2 v.8.2.12 (Stamatakis 2014). Prior to the BI analyses, the best-fit nucleotide substitution models for each DNA marker were selected using the Akaike Information Criterion (AIC) in jModelTest 2.1.6 (Darriba et al. 2012) via the CIPRES Gateway. The selected models were GTR + I + Γ for ITS and GTR + Γ for ETS. BI analyses were conducted in MrBayes with two parallel Markov Chain Monte Carlo (MCMC) runs for 20 million generations, each starting with a random tree and sampling one tree every 1,000^th^ generation. Tracer 1.7.2 (Rambaut et al. 2018) was used to assess parameter convergence and ensure effective sample size (ESS) values were ≥ 200. The first 25% of sampled trees were discarded as burn-in, and the remaining trees were used to compute posterior probabilities (PP) and construct a 50% majority-rule consensus tree. For the ML analyses, a partitioned model (-q) was applied with 1,000 bootstrap replicates (-#|-N), followed by a search for the best-scoring ML tree in a single run (-f a). The resulting trees were visualized and annotated using TreeGraph 2 (Stöver and Müller 2010).

## ﻿Results and discussion

**Figure 1.**
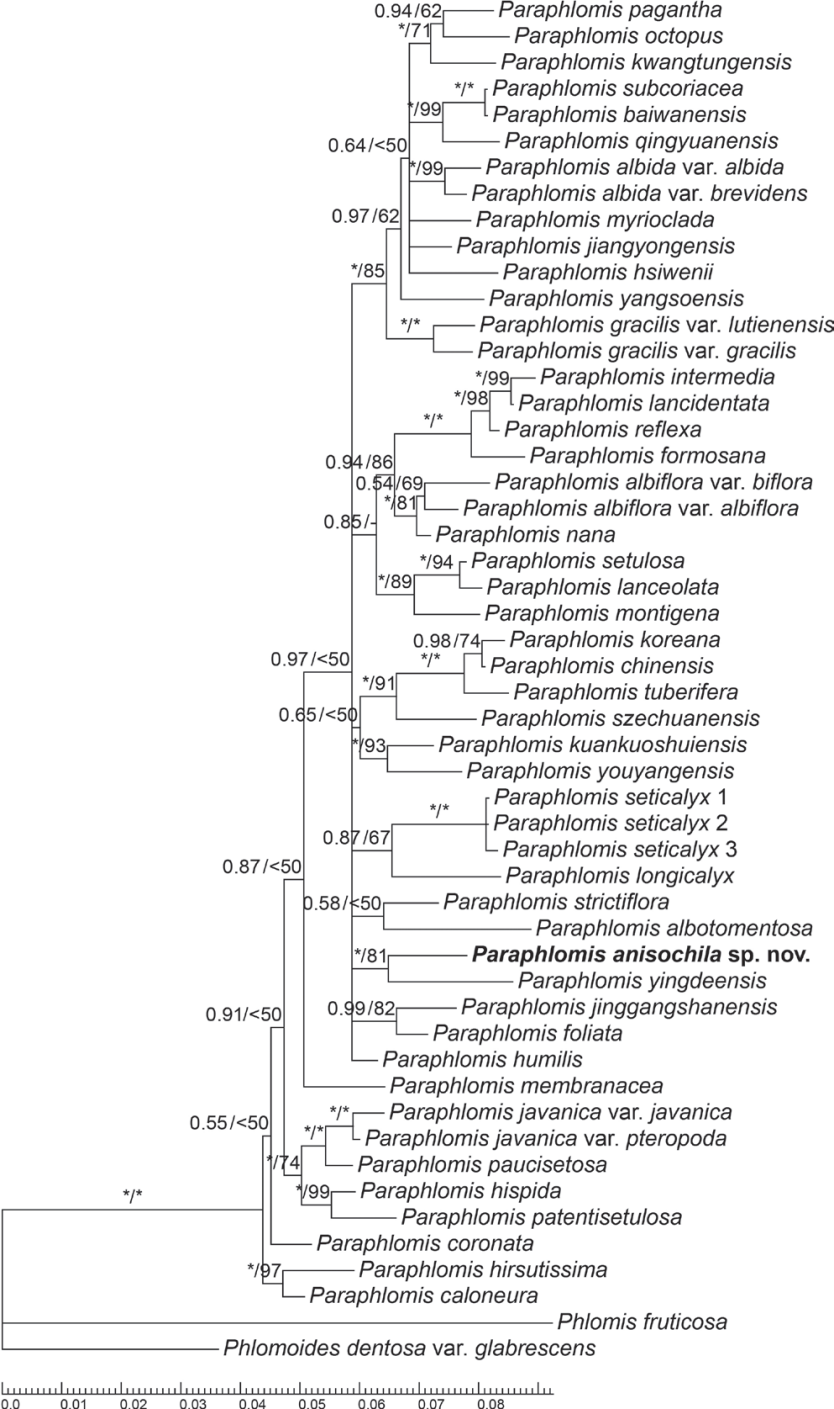
Bayesian 50% majority-rule consensus tree of *Paraphlomis* based on the combined nuclear (ITS and ETS) dataset. Support values ≥ 0.50 PP or 50% BS are displayed above the branches. An “*” indicates a support value of 1.00 PP or 100% BS and a “-” indicates a conflicting node in the BI and ML trees.

The aligned nrDNA dataset had a total length of 1,263 bp, comprising 815 bp for ITS and 448 bp for ETS. BI and ML analyses yielded largely congruent topologies, with only minor discrepancies at poorly supported nodes. Since the BI trees provided slightly better resolution, only the BI topology is presented (Fig. 1), with bootstrap support (BS) values from the ML analysis shown alongside the corresponding PP values.

The nrDNA tree topology (Fig. 1) was largely consistent with that reported by Chen et al. (2021) and Yuan et al. (2024). However, due to the limited number of informative sites, the nuclear phylogeny exhibited low resolution, with many nodes collapsing into polytomies. The new species was strongly supported as sister to *P.yingdeensis* W.Y. Zhao, Y.Q. Li & Q. Fan (Fig. 1: PP = 1.00, BS = 81%). While our morphological comparisons indicated that the new species was most similar to *P.seticalyx*, phylogenetic analyses revealed that the three accessions of *P.seticalyx* formed a well-supported clade (Fig. 1: PP = 1.00, BS = 100%) sister to *P.longicalyx* Y.P. Chen & C.L. Xiang (Fig. 1: PP = 0.87, BS = 67%). Both the *P.anisochila*-*P.yingdeensis* clade and the *P.seticalyx*-*P.longicalyx* clade were distinct lineages within *Paraphlomis*, but their relationships with other clades remained unresolved (Fig. 1). In Yuan’s (2024) unpublished species tree of *Paraphlomis*, reconstructed using 3,781 low-copy nuclear orthologs, the sister relationships between *P.anisochila* and *P.yingdeensis*, as well as between *P.seticalyx* and *P.longicalyx*, were also strongly supported. However, the two clades were inferred to be distantly related within the genus.

**Figure 2.**
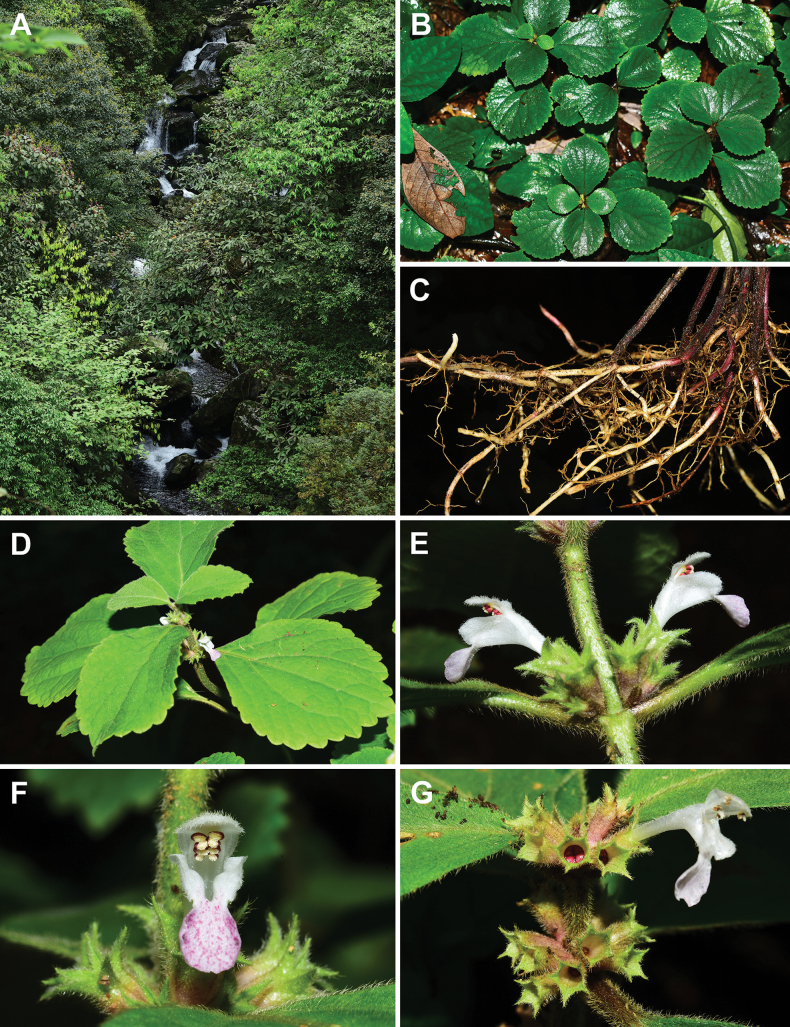
*Paraphlomisanisochila* from the type locality. **A.** Habitat; **B.** Habit; **C.** Stolons; **D.** Inflorescence; **E.** Lateral view of flowers; **F.** Frontal view of flower; **G.** Frontal view of calyces (Photographed by A. Liu).

**Figure 3.**
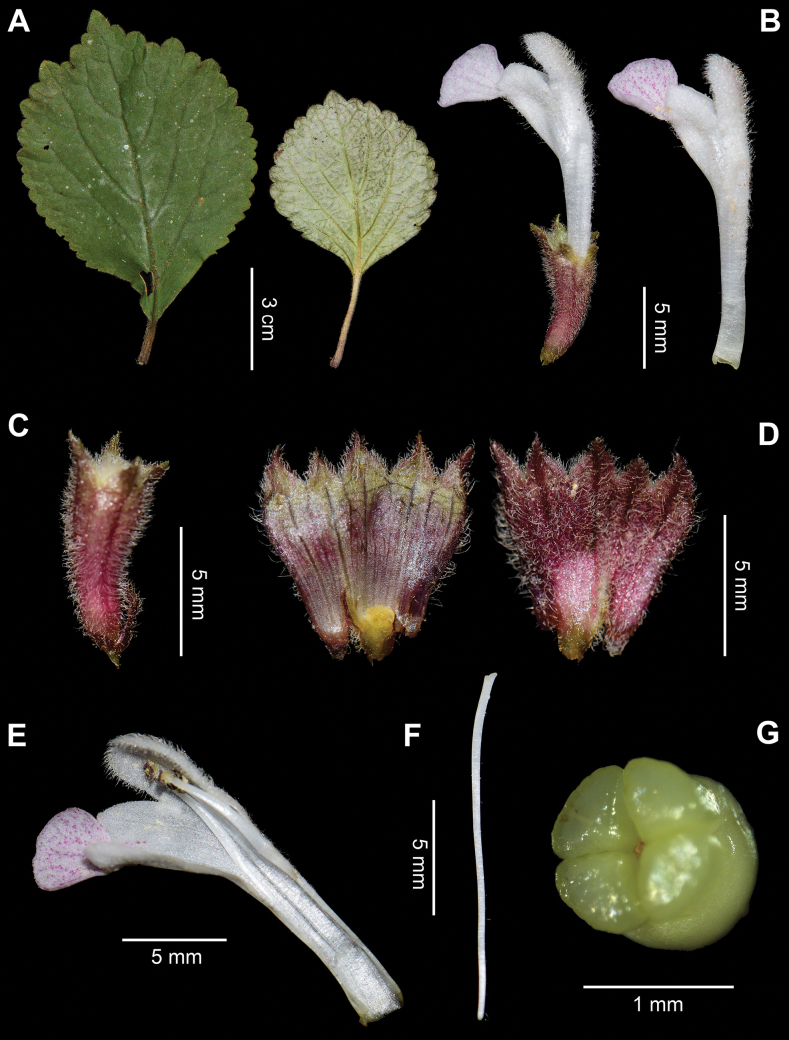
Foliar and floral morphology of *Paraphlomisanisochila*. **A.** Leaves; **B.** Flower and corolla; **C.** Calyx; **D.** Dissected calyces; **E.** Dissected corolla; **F.** Style; **G.** Ovary (Photographed by Y.P. Chen).

Morphologically, *P.anisochila* is a distinct species within the genus, characterized by its significantly unequal upper and lower corolla lips, with the upper lip being approximately half the length of the lower lip (Figs 2, 3). This trait sets it apart from most other *Paraphlomis* species, which typically possess subequal or equal corolla lips. The close relationship between *P.anisochila* and *P.yingdeensis* is reflected in their similar lamina shape, which is obovate to suborbicular, but the two species can be readily distinguished by their habit, indumentum, calyx length and apex, as well as corolla morphology (Table 1). Specifically, *P.yingdeensis* is a dwarf herb (10–20 cm tall) with densely villose stems and laminae, whereas *P.anisochila* is a taller herb (20–30 cm tall) with densely strigose stems and laminae. The apex of the calyx teeth is acuminate in *P.anisochila*, while it is bristle-like-acuminate in *P.yingdeensis*. Additionally, *P.yingdeensis* has yellow corollas without spots, in contrast to the white corollas of *P.anisochila*, which are often dotted with purple spots on the lower lip.

**Table 1.** Morphological comparisons among *Paraphlomisanisochila*, *P.seticalyx*, and *P.yingdeensis*.

Characters	*Paraphlomisanisochila*	*P.seticalyx*	*P.yingdeensis*
Habit	Herbs 20–30 cm tall	Herbs 20–60 cm tall	Herbs 10–20 cm tall
Stem	Densely strigose	Densely to sparsely strigose	Densely villous
Lamina	4–10 cm long, 4–8.5 cm wide, obovate or subcircular, base cuneate to broadly cuneate, apex obtuse, abaxially sparsely strigose on veins	10–12 cm long, 6–8 cm wide, broadly ovate, base broadly cuneate, subrounded to shallowly cordate, apex acute, abaxially sparsely strigose on veins	6.2–16.5 cm long, 4–11.5 cm wide, obovate or subcircular, base broadly cuneate to subrounded, apex obtuse, abaxially densely villous
Petiole	0.5–2.5 cm long, densely strigose	3.5–9 cm long, sparsely strigose	0.3–2.5 cm long, densely villous
Calyx	Green to purplish green, ca. 8 cm long, densely strigose outside, teeth ca. 2 mm long, apex acuminate	Light green or purplish green, ca. 1.2 cm long, densely strigose outside, teeth 3–4 mm long, apex acuminate	Light green, ca. 1.1 cm long, densely villous outside, teeth ca. 4 mm long, apex bristle-like-acuminate
Corolla	White, upper lip ca. 4 mm long, lower lip 7–8 mm long, dotted with purple spots, lateral lobes subcircular	White, upper lip ca. 7 mm long, lower lip ca. 7 mm long, dotted with purple spots, lateral lobes oblong	Yellow, upper lip ca. 6 mm long, lower lip ca. 7 mm long, without spots, lateral lobes oblong

Although *P.anisochila* is phylogenetically distantly related to *P.seticalyx*, the two species exhibit notable morphological similarities, including overall habit, indumentum, calyx morphology, and corolla color (Table 1). Except for the differences in corolla lips mentioned above, they also differ in lamina shape: *P.anisochila* has an obovate to suborbicular lamina with an obtuse apex and a cuneate to broadly cuneate base, whereas *P.seticalyx* possesses a broadly ovate lamina with an acute apex and a broadly cuneate to shallowly cordate base. Moreover, *P.anisochila* has significantly shorter petioles and calyces compared to *P.seticalyx*. The lateral lobes of the lower corolla lip are subcircular in *P.anisochila*, further differentiating the new species from *P.seticalyx* which has oblong lobes.

**Figure 4.**
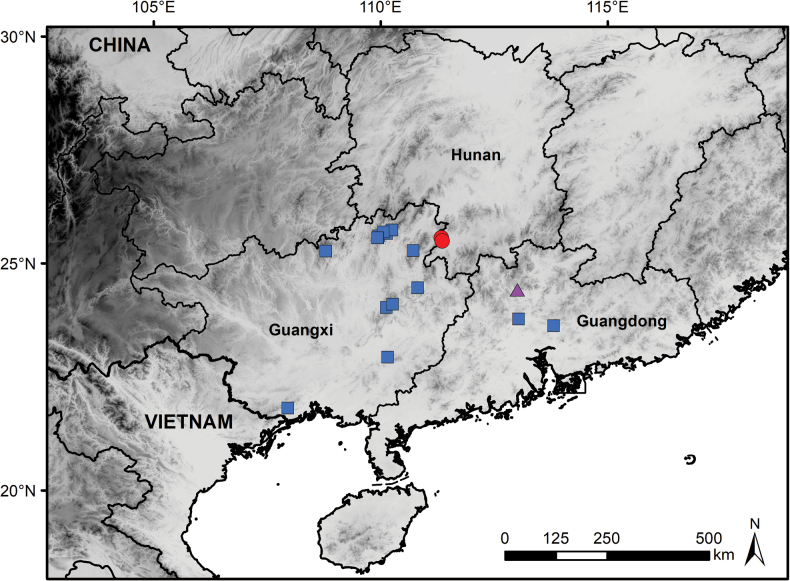
Distribution of *Paraphlomisanisochila* (red circles), *P.seticalyx* (blue squares), and *P.yingdeensis* (purple triangle).

Geographically, *P.anisochila*, *P.seticalyx*, and *P.yingdeensis* are all distributed within the EBLFs of southern China. *Paraphlomisanisochila* is currently known only from the border region between Hunan and Guangxi, whereas *P.yingdeensis* is restricted to the karst landscapes of northern Guangdong (Fig. 4; Guo et al. 2023). In contrast, *P.seticalyx* has a broader distribution, primarily in eastern Guangxi, with recent records extending into central Guangdong (Fig. 4; Zeng et al. 2024). The relatively disjunct distribution of *P.anisochila* and *P.yingdeensis*, despite their close phylogenetic relationship, suggests potential habitat differentiation, historical fragmentation, or the presence of unrecognized populations in the intervening regions.

### ﻿Taxonomic treatment

#### ﻿ Paraphlomis anisochila

Taxon classificationPlantaeLamialesLamiaceae

Y.P.Chen, A.Liu & F.Zhang sp. nov.

56677D5B-CCEE-5B85-AF20-2E4DE1472B98

urn:lsid:ipni.org:names:77365181-1

Figs 2 , 3 , Appendix 2

##### Type.

China. Hunan: • Daoxian County, Qingtang Town, Dupangling Natural Reserve, Jinliyuan, 25°29'09"N, 111°21'31"E, elev. 547 m, 2 Aug. 2020, *A. Liu et al. LK1088* (holotype: KUN1643901!; isotypes: CSFI075346!, CSH!, KUN1643902!).

##### Diagnosis.

*Paraphlomisanisochila* is morphologically similar to *P.seticalyx* and *P.yingdeensis*, but differs from the former in having an obovate to subcircular (vs. broadly ovate) lamina, 0.5–2.5 cm (vs. 3.5–9 cm) long petioles, unequal (vs. subequal) corolla lips, and subcircular (vs. oblong) lateral lobes of the lower corolla lip, from the latter in having an abaxially sparsely strigose (vs. densely villous) lamina, an acuminate (vs. bristle-like-acuminate) apex of calyx tooth, and a white (vs. yellow) corolla with unequal (vs. subequal) lips.

Herbs perennial, 20–30 cm tall, erect, stoloniferous. Stems 4-angled, green to purplish-red, densely retrorse strigose, leafless toward base. Leaves opposite; lamina obovate to subcircular, thick papery, 4–10 cm long, 4–8.5 cm wide, base cuneate to broadly cuneate, apex obtuse, margin crenate-serrate, adaxially green, densely appressed strigose, abaxially light green, sparsely glandular, sparsely strigose on veins; lateral veins 3–4-paired; petioles 0.5–2.5 cm long, densely retrorse strigose. Verticillasters many-flowered, globose, ca. 4 cm in diam.; bracteoles lanceolate to subulate, 3–5 mm long, densely strigose. Calyx green to purplish red, ca. 8 mm long, densely strigose outside, densely strigose at apex inside, 10-veined; teeth 5, subequal, triangular, ca. 2 mm long, 2–3 mm wide, apex acuminate. Corolla white, 2–2.1 cm long, densely villose outside; tube ca. 1.3 cm long, ca. 1.5 mm wide, pubescent annulate inside at 1/3 distance from base; 2-lipped, upper lip oblong, entire, erect, concave, ca. 4 mm long, ca. 3 mm wide; lower lip spreading, 7–8 mm long, ca. 6 mm wide, 3-lobed, medium lobe largest, subcircular, dotted with purple spots, ca. 3.5–4.5 mm long, ca. 4 mm wide, lateral lobes semicircular, ca. 1.5 mm long, ca. 3 mm wide. Stamens 4, straight, included, filaments flat, glabrous, anther cells 2, parallel. Style included, glabrous, apex inconspicuously 2-lobed. Ovary glabrous, apex truncate. Mericarps not seen.

##### Phenology.

Flowering from June to August.

##### Etymology.

The specific epithet “*anisochila*” refers to the distinct corolla morphology of the new species, characterized by significantly unequal upper and lower lips, which differentiates it from other *Paraphlomis* species that typically possess subequal/equal corolla lips.

##### Distribution and habitat.

The new species is currently known only from the Dupangling Natural Reserve in southwestern Hunan Province, China, where it typically inhabits EBLFs along streams at elevations ranging from 550 to 750 m (Fig. 4).

##### Chinese name

**(assigned here).** yì chún jiǎ cāo sū (异唇假糙苏).

##### Additional specimens examined.

China. Hunan: • Daoxian County, Shouyan Town, Kongshuyan Village, Dupangling Natural Reserve, 25°33'29"N, 111°20'26"E, elev. 748 m, 24 Jun. 2020, *A. Liu LK0932* (CSFI!, KUN!).

##### Specimens of *P.seticalyx* examined.

China. Guangdong: • Qingyuan City, Qingxin District, 16 Jul. 2018, *Y. Tong et al. TY20080901* (KUN!); • Qingyuan City, Qingxin District, Mt. Bijia, 23°46'19"N, 113°02'13"E, elev. 120 m, among rocks at roadside, 1 Jun. 2023, *Q.G. Zeng s.n.* (KUN!). Guangxi: • Lo-mong, 24 Jun. 1931, *S.S. Sin et al. 22331* (holotype: IBSC0005129!); • Ku-chun, 29 Jun. 1931, *S.S. Sin et al. 21627* (IBSC0585111!); • ibid., 26 Jun. 1934, *S.S. Sin et al. 23298* (IBSC0585110!); • Dayao Mountains, elev. 960 m, 30 Jun. 1958, *Y.K. Li 400437* (IBK00059955!, IBSC0585115!); • ibid., 9 Aug. 1958, *Y.K. Li 400943* (IBK00059954!, IBSC0585114!); • Fangchenggang City, Fulong Town, Mt. Pinglong, Masheniao Rainfall, at streamside, elev. 590 m, 10 Jul. 2020, *Shiwandashan Exped. 2739* (IBK00234568!); • Guiping City, Shepo Town, 11 Jun. 1978, *B.Q. Yang 8-02796* (GXMI053292!); • Jinxiu County, Changdong Town, Dishui Village, 10 Jun. 1977, *Jinxiu Exped. 5-1-83* (GXMI053293!); • Jinxiu County, Gonghe Village, elev. 1020 m, 16 Sept. 1981, *Dayaoshan Exped. 810484* (GXMI053294!); • Jinxiu County, Laoshan, 23 Jul. 1985, *J.Y. Luo 65435* (GXMI053295!); • Lingchuan County, Haiyang Town, Antai Village, Baidaidi, 25°16'50.8"N, 110°42'59.6"E, elev. 513 m, 19 Jun. 2013, *Lingchuan Exped. 450323130619027LY* (GXMG0148849!, IBK00400020!); • Lingchuan County, Lantian Town, Jiangzhouping, 25°39'22.2"N, 110°7'57.6"E, elev. 1020 m, 2 Jul. 2013, *Lingchuan Exped. 450323130702006LY* (GXMG0148848!, IBK00400021!); • Lingchuan County, Qingshitan Town, Honglingtou Village, Zongshuping, 25°43'52.89"N, 110°14'54.78"E, elev. 609 m, 8 Aug. 2013, *Lingchuan Exped. 450323130808002LY* (GXMG0148847!, IBK00400019!); • Lingui District, Wantian Town, Hongtan, in forests, elev. 830 m, 29 Oct. 2023, *A. Liu et al. GX06* (KUN!); • Longsheng County, Dadi Town, at streamside in forest, elev. 800 m, 11 Jul. 1955, *Guangfu Exped. 00873* (IBK00191748!, IBSC0585112!, KUN0277040!, NAS00224462!, PE00834807!); • Longsheng County, Guangfu, Mt. Maozhu, elev. 1020 m, 19 Jul. 1955, *Guangfu Exped. 00645* (IBK00191747!, IBSC0585113!, NAS00224461!, PE00834808!); • Longsheng County, Mt. Tianping, 22 Jun. 1957, *H.F. Qin & Z.T. Li 70422* (IBK00059952!, IBSC0585117!); • Longsheng County, Huaping, near Hongtan, elev. 920 m, 14 Sept. 1984, *Y.Z. Wei & Q.H. Lv 20352* (IBK00059953!); • Longsheng County, Huaping Natural Reserve, Weiqingling, elev. 1820 m, 30 Oct. 2006, *Huaping Exped. H1200* (IBK00228432!); • Longsheng County, Heping Town, 25°41'26"N, 110°03'25"E, elev. 542 m, 16 Apr. 2013, *Longsheng Exped. 450328130416014LY* (GXMG0130436!, IBK00362590!); • Longsheng County, Huaping National Natural Reserve, 25°33'47.25"N, 109°56'21.98"E, elev. 1338 m, 3 Sept. 2013, *Longsheng Exped.* 450328130903029LY (GXMG0130437!, IBK00362591!); • ibid., 25°33'37"N, 109°55'52"E, elev. 1645 m, 29 Aug. 2014, *Longsheng Exped. 450328140829002LY* (GXMG0130438!, IBK00362560!); • Longsheng County, Sishui Town, Liluo Forest Farm, Xijiangping Reservoir, elev. 950 m, 22 Jul. 2015, *W.B. Xu & S.M. Xiong 12386* (IBK!); • Luocheng County, in forest, 31 May 1989, *Beijing Exped. 895418* (PE00834811!); • ibid., elev. 500 m, 1 Jun. 1989, *Beijing Exped. 895679* (PE00834812!); • Rongshui County, Wangdong Town, Jiuwanshan Natural Reserve, elev. 500 m, 10 Jul. 1958, *S.H. Chun 14852* (IBK00370418!, IBSC0585116!, KUN0228149!, NAS00072430!, PE00834805!).

##### Specimens of *P.yingdeensis* examined.

China. Guangdong: • Yingde City, Boluo Town, on the way from Xinzhai Village to Changshan Village, on the limestone cliff, 24°24'N, 113°0'E, elev. 61 m, 29 May 2021, *W.Y. Zhao et al. ZWY-2092* (isotype: KUN!).

## Supplementary Material

XML Treatment for Paraphlomis anisochila

## Figures and Tables

**Figure A1.**
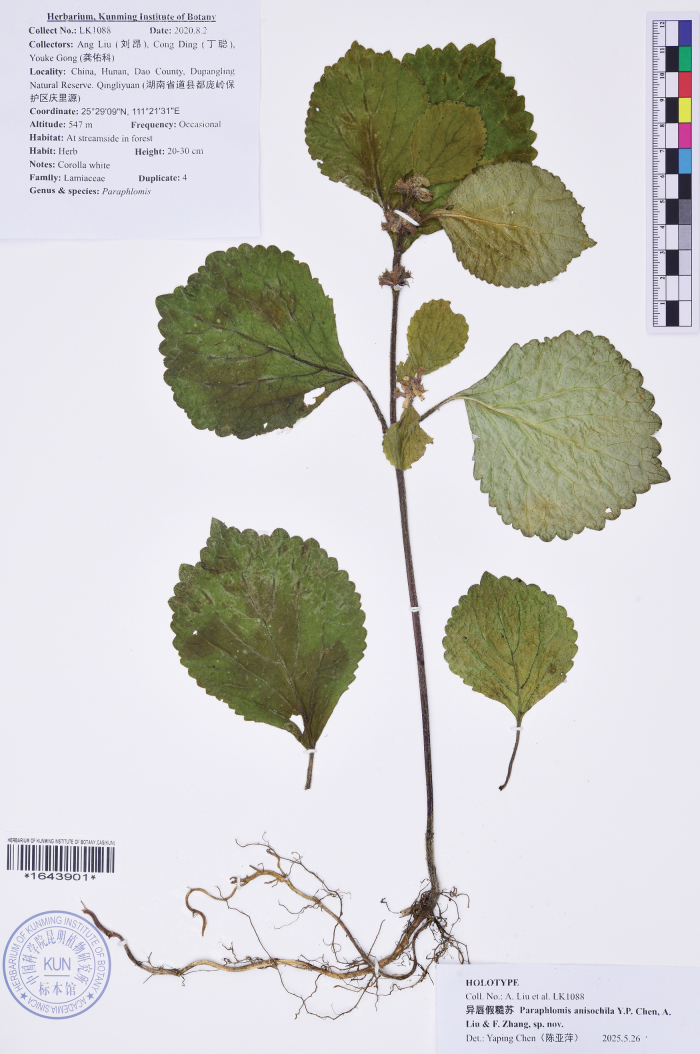
Holotype specimen of *Paraphlomisanisochila*.

## ﻿Additional information

### Use of AI

No use of AI was reported.

### Author ORCIDs

Ang Liu https://orcid.org/0000-0001-6281-7145

Fan Zhang https://orcid.org/0009-0007-3947-128X

Ya-Ping Chen https://orcid.org/0000-0002-7502-1848

### Data availability

Newly generated sequences in this study were deposited in the NCBI database.

## ﻿Appendix 1

Specimen information (taxon, voucher, herbarium, country) for samples newly sequenced in the present study with GenBank accession numbers for ITS and ETS. Herbarium abbreviations are listed after the vouchers. An “*” indicates a sequence newly generated, while a “-” indicates missing data. Only GenBank accession numbers are listed for sequences downloaded from NCBI.

***Paraphlomisalbida*** Hand.-Mazz. var. ***albida***, MW602124, MW602091; ***Paraphlomisalbida*** var. ***brevidens*** Hand.-Mazz., MW602130, MW602098; ***Paraphlomisalbiflora*** (Hemsl.) Hand.-Mazz. var. ***albiflora***, -, MW602101; ***Paraphlomisalbiflora*** var. ***biflora*** (Y.Z. Sun) C.Y. Wu, OR594010, OR576847; ***Paraphlomisalbotomentosa*** C.Y. Wu, OR593999, OR576835; ***Paraphlomisanisochila*** Y.P. Chen, A. Liu & F. Zhang, A. Liu et al. LK1088 (KUN), 2 Aug. 2020, Hunan, China, PV476762*, PV478009*; ***Paraphlomisbaiwanensis*** W.Y. Zhao, Y.P. Chen & Q. Fan, PP897029, PP897950; ***Paraphlomiscaloneura*** K.J. Yan, Y.P. Chen & Y.F. Huang, OQ627454, OQ628080; ***Paraphlomischinensis*** (Benth.) J.C. Yuan, Y.P. Chen & C.L. Xiang, MW602147, MW602117; ***Paraphlomiscoronata*** (Vaniot) Y.P. Chen & C.L. Xiang, MW602137, MW602107; ***Paraphlomisfoliata*** (Dunn) C.Y. Wu & H.W. Li, -, MW602097; ***Paraphlomisformosana*** (Hayata) T.H. Hsieh & T.C. Huang, OR594007, OR576844; ***Paraphlomisgracilis*** (Hemsl.) Kudô var. ***gracilis***, MW602141, MW602111; ***Paraphlomisgracilis*** var. ***lutienensis*** (Y.Z. Sun) C.Y. Wu, MW602131, MW602099; ***Paraphlomishirsutissima*** C.Y. Wu & H.W. Li, OQ627453, OQ628079; ***Paraphlomishispida*** C.Y. Wu, MW602132, MW602102; ***Paraphlomishsiwenii*** Y.P. Chen & Xiong Li, OP605346, OP609841; ***Paraphlomishumilis*** (Miq.) J.C. Yuan,Y.P. Chen & C.L. Xiang, -, OR576876; ***Paraphlomis intermedia C.Y. Wu & H.W. Li***, MW602135, MW602105; ***Paraphlomisjavanica*** (Blume) Prain var. ***javanica***, MW602121, MW602088; ***Paraphlomisjavanica*** var. ***pteropoda*** D. Fang & K.J. Yan, MW602140, MW602110; ***Paraphlomisjiangyongensis*** X.L. Yu & A. Liu, MW602128, MW602095; ***Paraphlomisjinggangshanensis*** Boufford, W.B. Liao & W.Y. Zhao, OR594005, OR576841; ***Paraphlomiskoreana*** S.C. Ko & G.Y. Chung, OQ834441, OQ848661; ***Paraphlomiskuankuoshuiensis*** R.B. Zhang, D. Tan & C.B. Ma, OR594021, OR576858; ***Paraphlomiskwangtungensis*** C.Y. Wu & H.W. Li, PP713070, PP706067; ***Paraphlomislanceolata*** Hand.-Mazz., MW602145, MW602115; ***Paraphlomislancidentata*** Y.Z. Sun, MW602136, MW602106; ***Paraphlomislongicalyx*** Y.P. Chen & C.L. Xiang, OK104771, OK104774; ***Paraphlomismembranacea*** C.Y. Wu & H.W. Li, -, MW602100; ***Paraphlomismontigena*** (X.H. Guo & S.B. Zhou) J.C. Yuan, Y.P. Chen & C.L. Xiang, OM836064, OM884453; ***Paraphlomismyrioclada*** J.C.Yuan, Y.P. Chen & C.L. Xiang, OR594042, OR576880; ***Paraphlomisnana*** Y.P. Chen, C. Xiong & C.L. Xiang, OM836062, OM884451; ***Paraphlomisoctopus*** Q. Fan, Y.P. Chen & Ying Liu, MW602126, MW602093; ***Paraphlomispagantha*** Dunn, OP605345, OP609840; ***Paraphlomispatentisetulosa*** C.Y. Wu, OQ627455, OQ628081; ***Paraphlomispaucisetosa*** C.Y. Wu, MW602125, MW602092; ***Paraphlomisqingyuanensis*** W.Y. Zhao, R.M. Wu & Q. Fan, PP897031, PP897952; ***Paraphlomisreflexa*** C.Y. Wu & H.W. Li, MW602122, MW602089; ***Paraphlomisseticalyx*** C.Y. Wu 1, OR594020, OR576857; ***Paraphlomisseticalyx*** C.Y. Wu 2, A. Liu et al. GX06 (KUN), 29 Oct. 2023, Guangxi, China, PV476763*, PV478010*; ***Paraphlomisseticalyx*** C.Y. Wu 3, Q.G. Zeng s.n. (KUN), 1 June 2023, Guangdong, China, PV476764*, PV478011*; ***Paraphlomissetulosa*** C.Y. Wu & H.W. Li, OR594001, OR576837; ***Paraphlomisstrictiflora*** J.C. Yuan, B. Chen & C.L. Xiang, -, OP609839; ***Paraphlomissubcoriacea*** C. Y. Wu ex H. W. Li, PP897033, PP897954; ***Paraphlomisszechuanensis*** (C.Y. Wu) J.C.Yuan, Y.P. Chen & C.L. Xiang, OR594014, OR576851; ***Paraphlomistuberifera*** (Makino) J.C.Yuan, Y.P. Chen & C.L. Xiang, OR594038, OR576875; ***Paraphlomisyangsoensis*** (Y.Z. Sun) J.C. Yuan, Y.P. Chen & C.L. Xiang, MW602142, MW602112; ***Paraphlomisyingdeensis*** W.Y. Zhao, Y.Q. Li & Q. Fan, OP605348, OP609843; ***Paraphlomisyouyangensis*** H. Jiang, R.B. Zhang & Tan Deng, OR488121, OR487461; ***Phlomisfruticosa*** L., MW602119, MW602086; ***Phlomoidesdentosa*** var. ***glabrescens*** (Danguy) C.L. Xiang & H. Peng, MW602120, MW602087.
